# Head morphology of *Tricholepidion gertschi *indicates monophyletic Zygentoma

**DOI:** 10.1186/1742-9994-11-16

**Published:** 2014-03-13

**Authors:** Alexander Blanke, Markus Koch, Benjamin Wipfler, Fabian Wilde, Bernhard Misof

**Affiliations:** 1 Zoologisches Forschungsmuseum Alexander Koenig, Zentrum für molekulare Biodiversitätsforschung, Adenauerallee 160, 53113 Bonn, Germany; 2 Institute of Evolutionary Biology and Animal Ecology, University of Bonn, An der Immenburg 1, 53121 Bonn, Germany; 3 Entomology Group, Institut für Spezielle Zoologie und Evolutionsbiologie, Friedrich-Schiller-Universität Jena, Erbertstraße 1, 07743 Jena, Germany; 4 Helmholtz-Zentrum Geesthacht, Zentrum für Material- und Küstenforschung GmbH, Max-Planck-Straße 1, 21502 Geesthacht, Germany

**Keywords:** Lepidotrichidae, Muscle equipment, MicroCT, Anatomy, Synchrotron

## Abstract

The relic silverfish *Tricholepidion gertschi *is the sole extant representative of the family Lepidotrichidae. Its phylogenetic position is of special interest, since it may provide crucial insights into the early phenotypic evolution of the dicondylian insects. However, the phylogenetic position of *T. gertschi *is unclear. Originally, it was classified among silverfish (Zygentoma), but various alternative relationships within Zygentoma as well as a sistergroup relationship to all remaining Zygentoma + Pterygota are discussed, the latter implying a paraphyly of Zygentoma with respect to Pterygota. Since characters of the head anatomy play a major role in this discussion, we here present the so far most detailed description of the head of *T. gertschi *based on anatomical studies by synchrotron micro-computer tomography and scanning electron microscopy. A strong focus is put on the documentation of mouthparts and the anatomy of the endoskeleton as well as the muscle equipment. In contrast to former studies we could confirm the presence of a *Musculus hypopharyngomandibularis *(0md4). The ligamentous connection between the mandibles composed of *Musculus tentoriomandibularis inferior *(0md6) is also in contact with the anterior tentorium. Phylogenetic analysis of cephalic data results in monophyletic Zygentoma including *T. gertschi*. Zygentoma are supported by the presence of a set of labial muscles originating at the postocciput, presence of an additional intralabral muscle, and four labial palpomeres. Character systems like the genitalic system, the mating behaviour, the segmentation of the tarsi, the overall body form, and the presence of ocelli which were proposed in other studies as potentially useful for phylogenetic reconstruction are evaluated.

## Introduction

**Figure 1:**
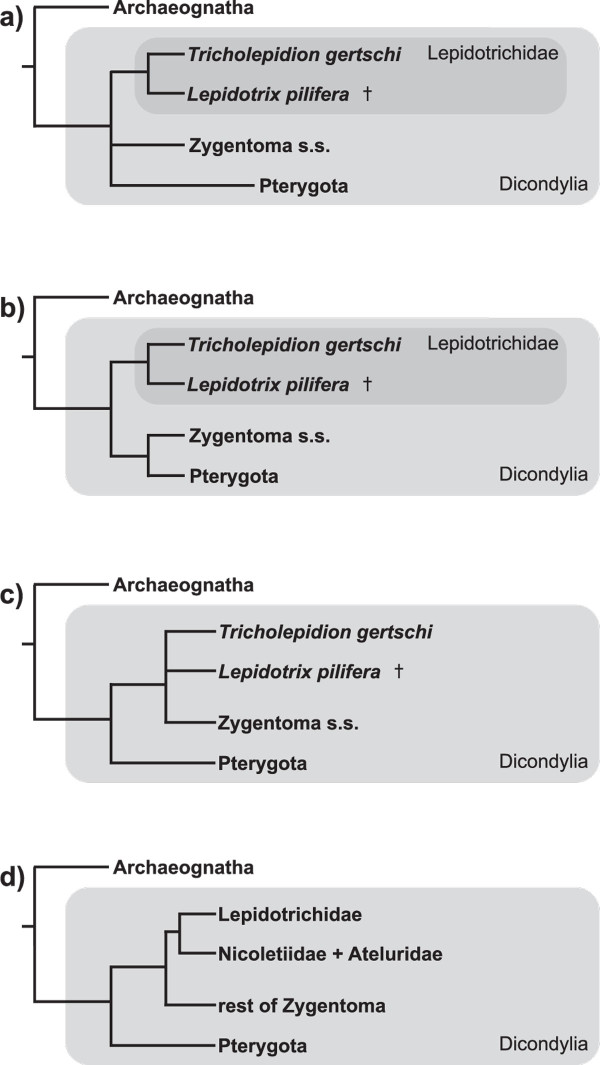
**Hypotheses concerning the phylogenetic position of *Tricholepidion gertschi***. **a) **the position of Lepidotrichidae remained unclear after analysis of cephalic characters [11,14] and characters of the whole body with a focus on attachment structures [13], **b) **Lepidotrichidae as the sistergroup to all remaining Dicondylia was hypothesized by Kristensen [15] and Stys & Zrzavý [16], **c) ***T. gertschi *as the sistergroup to the remaining Zygentoma, with unclear resolution of †*L. pilifera*, was hypothesized by Koch [17] and Engel [18], **d) **Lepidotrichidae as closely related to Nicoletiidae (+ Ateluridae) within Zygentoma was hypothesized by Wygodzinsky [10] and is supported by sperm characters according to Dallai et al. [19].

The relic silverfish *Trichlepidion gertschi *occurs only in the coastal region of northern California. The species is characterized by a number of peculiarities with respect to all other extant silverfish species (= Euzygentoma) such as the presence of ocelli (in addition to compound eyes), 5-segmented tarsi [1], styli and coxal vesicles on almost all pregenital segments of the abdomen, and a ligamentous head endoskeleton. Its phylogenetic position with Zygentoma or Dicondylia is unclear (for an overview see [1,2]). Molecular [3-7] and morphological studies [8-13] disagree whether *T. gertschi *is the sister species to all other dicondylians (Zygentoma + Pterygota), sister species to all other Zygentoma, or a subgroup within Zygentoma (Figure 1).

However, the phylogenetic position of *T. gertschi *is crucial to understand the evolution of several morphological characters in the stem lineage of Dicondylia, e.g. presence of a proventriculus [10], sperm configuration [20,21], the general organisation of the head [11,14] and thorax [22] including muscle equipment, and the composition of ovaries [10].

Available cephalic data did not help thus far to resolve the phylogenetic relationships of *T. gertschi *to remaining Zygentoma [11,14]. This contribution aims to augment the analyses of the head character system by providing a detailed description of the head morphology of *T. gertschi *with a strong focus on the documentation of mouthparts, endoskeleton and muscle equipment. We show that characters of the cephalic morphology indeed provide information on a sistergroup relationship of *T. gertschi *and the remaining studied Zygentoma.

## Materials and methods

All specimens were fixed in Bouin's solution [23] and investigated using *s*ynchrotron *r*adiation *micro*-*c*omputer *t*omography (SR-μCT), so that they could be used to complement the character matrix (see additional files 1,2,3) used for phylogenetic reconstruction. The species studied include *Tricholepidion gertschi *Wygodzinski 1961 (Lepidotrichidae), *Thermobia domestica *(Packard, 1873) (Lepismatidae), *Lepisma saccharina *Linneaus, 1758 (Lepismatidae), and *Atelura formicaria *Heyden, 1855 (Nicoletiidae). The outer anatomy of *T. gertschi *was furthermore investigated with scanning electron microscopy (SEM).

SR-μCT was done using the recommendations of Betz et al. [24] and the respective beamline staff. Prior to scanning, the sample was critical point dried (CPD) (Model E4850, BioRad), mounted on specimen holders, and put into the scan chamber several hours in advance to allow for temperature acclimatisation which avoids movement artefacts. Scanning was performed at the Paul-Scherrer Institute (PSI, Villigen, Switzerland) with a stable energy beam of 8.5 keV in attenuation mode, 10× magnification, 500 ms exposure time, and 1601 projections within 180° (see [25] for the beamline configuration) and at the Deutsches Elektronen Synchrotron (DESY, Hamburg, Germany) at the beamline DORIS III/BW2 and beamline PETRA III/IBL P05 with a stable energy of 8 keV with high density resolution [26] in attenuation contrast mode. The field of view, exposure time, and number of projections was adjusted for each specimen.

Segmentation of structures and rendering of the resulting 3D model was performed with the software packages Reconstruct [27] and Blender (http://www.blender.org). Both software packages are distributed under the general public license (GPL). Tables and figures were edited with GIMP ver. 2.8, Inkscape ver. 0.48 and Scribus ver. 1.4.1 (all GPL). A 3D model of the head of *T. gertschi *is available (additional file 4) which facilitates identification of internal structures. Please download the software Blender to view the model. Additionally, transverse sections of the head are provided as a film sequence (in AVI format; see additional file 5)

For scanning electron microscopy (SEM) the specimen was transferred in a series of steps into 100% ethanol, critical point dried (Model E4850, BioRad), and sputter coated (Model Anatech Hummer VII). Microscopy was performed on a Hitachi S-2460 N scanning electron microscope using a rotatable sample holder [28]. The terminology of skeletal elements follows Seifert [29], the muscular one Wipfler et al. [30].

### Phylogenetic analyses

Parsimony analyses of 139 cephalic characters (see Appendix) and Bremer support (BR) calculations were carried out with TNT [31] using 1,000 heuristic searches starting with random addition of taxa (Wagner trees; Tree-Bisection-Reconnection (TBR) branch swapping, with 100 trees saved per replicate). All characters were equally weighted and unordered. The archaeognathan *Machilis germanica *was selected as the outgroup. Only unambiguous changes were mapped on the optimal trees. Character numbers and states are given in brackets using the following syntax: (character number : character state). The character matrix is derived from Blanke et al. [32] and is based on the matrix of Wipfler et al. [30]. Please refer to the electronic supplement (additional file 6) for a complete tree showing all taxa.

## Results

### External head capsule

**Figure 2:**
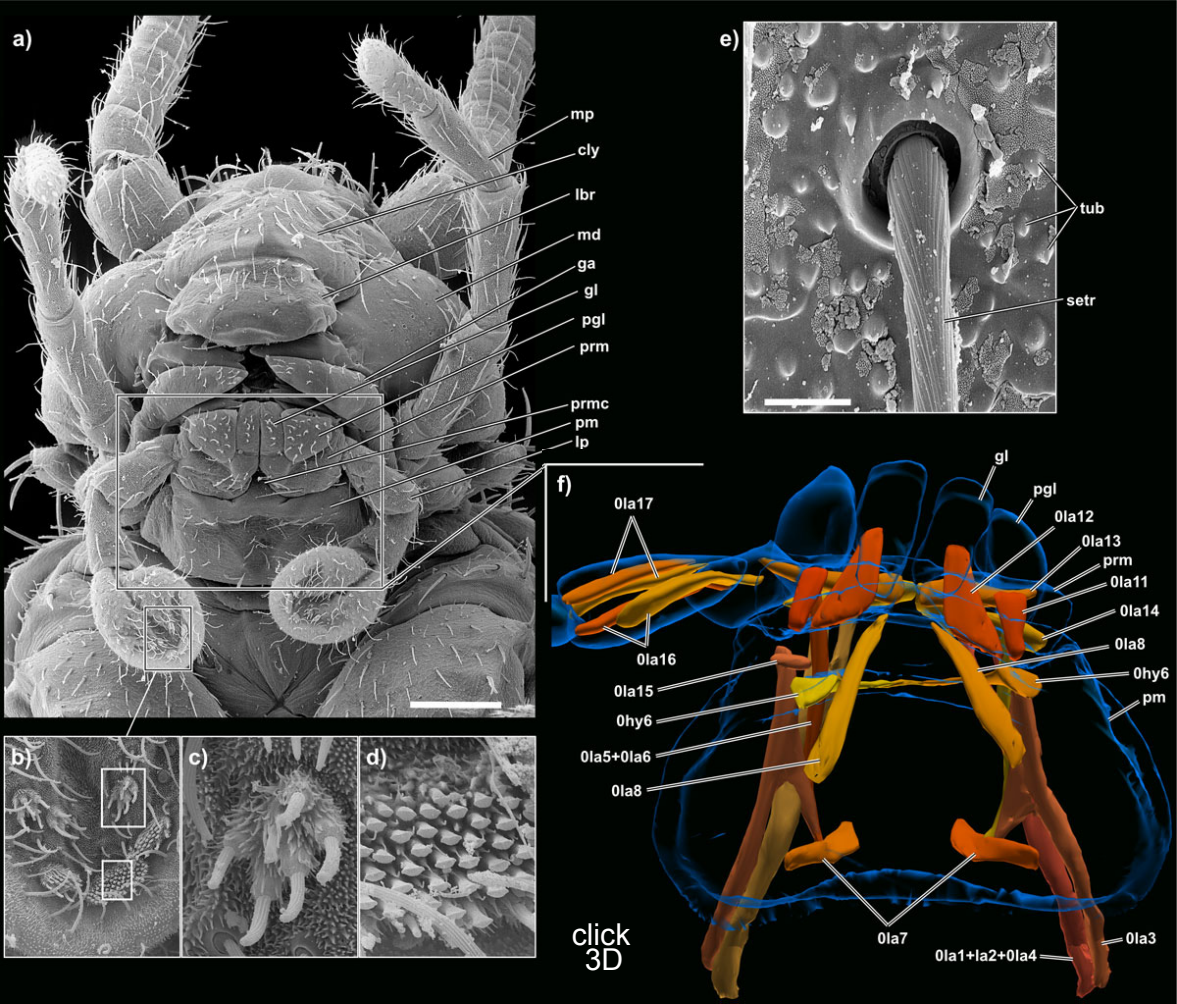
**SEM micrographs and 3D reconstruction of *Tricholepidion gertschi*;**. **a) **ventral overview of the head; scale bar 200 μm **b) **detail of the ventral side of the fourth labial palpus segment; **c) **detail of the first sensillum type; **d) **detail of the second sensillum type; **e) **detail of a trichoid sensillum; scale bar 5 μm f) labial musculature in ventral view. Abbreviations: cly, clypeus; ga, galea; gl, glossa; lbr, labrum; lp, labial palpus; md, mandible; mp, maxillar palpus; pgl, paraglossa; pm, postmentum; prm, prementum; prmc, premental cleft; setr, trichoid sensillum; tub, tubercles. Please click on the figure to activate the 3D content. For muscle references see main text. Images not to scale to each other.

**Figure 3:**
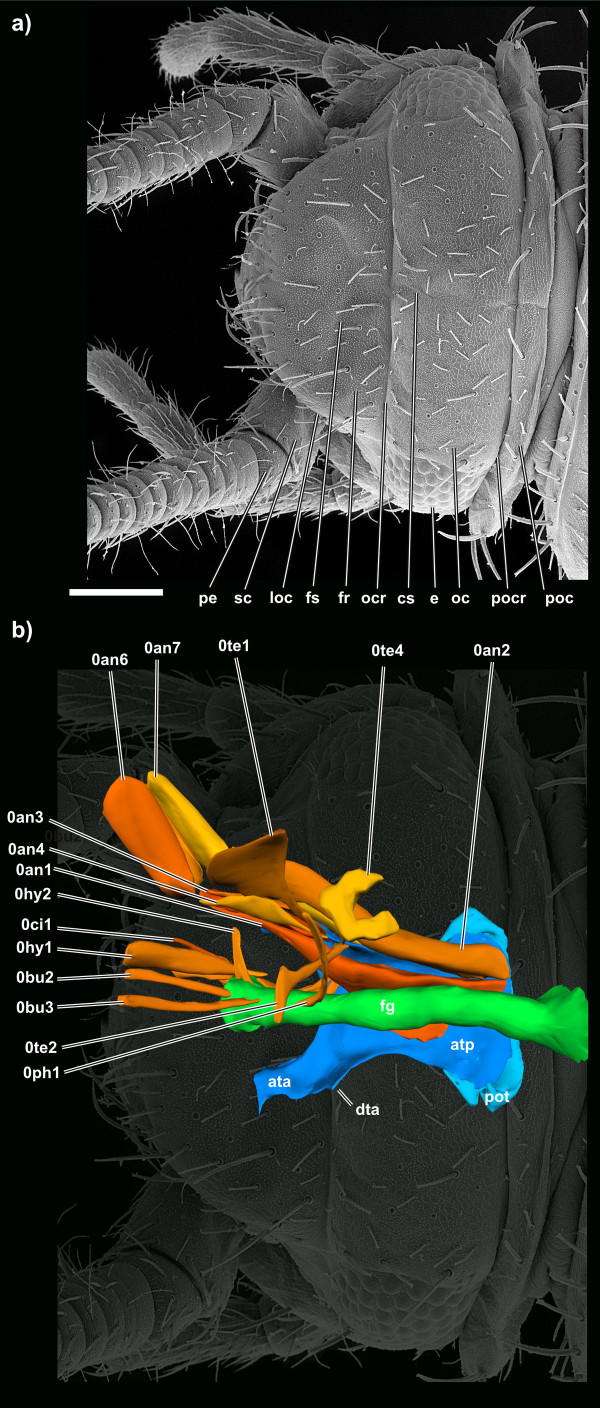
**SEM micrographs and 3D reconstruction of *Tricholepidion gertschi*;**. **a) **dorsal overview of the head; **b) **the musculature of the antenna, cephalic digestive tract, and tentorium in dorsal view. Abbreviations: ata, anterior tentorial arm; atp, anterior tentorial plate; cs, coronal suture; dta, dorsal tentorial arm; e, eye; fg, foregut; fr, frons; fs, frontal suture; loc, lateral ocellus; oc, occiput; ocr, occipital ridge; pe, pedicellus; poc, postocciput; pocr, postocciptial ridge; sc, scapus; Scale bar 200 μm. Images not to scale to each other. For muscle references see main text.

The orthognathous head (Figure 2a) bears numerous sensilla. Trichoid sensilla (= setae) are up to 100 μm long, directed anteriorly and occur with a density of 10-12 sensilla per 100 μm^2^. Among them, numerous (>500/100 μm^2^) small tubercles on the exoskeleton, possibly sensorial in function, cover the entire head (Figure 2e). Both sensillum types also occur on certain regions of the mouthparts (details see below). The compound eyes, containing ~40 ommatidia, are positioned immediately behind the antennae and dorsal to the externally visible posterior mandibular articulation (Figure 3a, 4b). The three ocelli are barely visible in SEM specimens while they are of whitish colour in living specimens. The middle ocellus lies centrally directly above the epistomal ridge, the lateral ocelli behind the antennal bases (Figures 3a, 5a). Their lenses do not protrude from the head.

**Figure 4:**
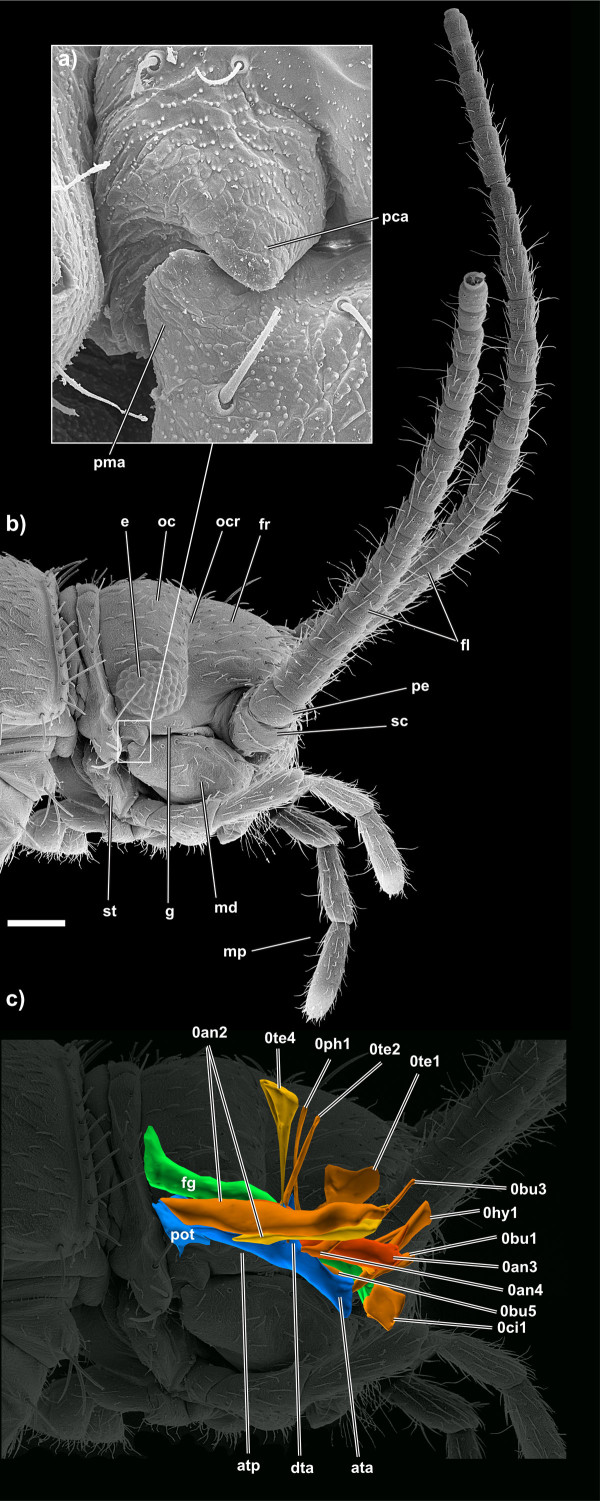
**SEM micrographs and 3D reconstruction of *Tricholepidion gertschi*;**. **a) **detail of the posterior mandibular articulation; **b) **lateral overview of the head; **c) **the musculature of the antenna, cephalic digestive tract, and tentorium in lateral view. Abbreviations: ata, anterior tentorial arm; atp, anterior tentorial plate; dta, dorsal tentorial arm; e, eye; fl, flagellum; fg, foregut; fr, frons; g, gena; md, mandible; mp, maxillary palpus; oc, occiput; ocr, occipital ridge; pca, posterior cephalic articulation; pe, pedicellus; pma, posterior mandibular articulation; pot, posterior tentorium; sc, scapus; st, stipes. Images not to scale to each other. Scale bar 200 μm. For muscle references see main text.

**Figure 5:**
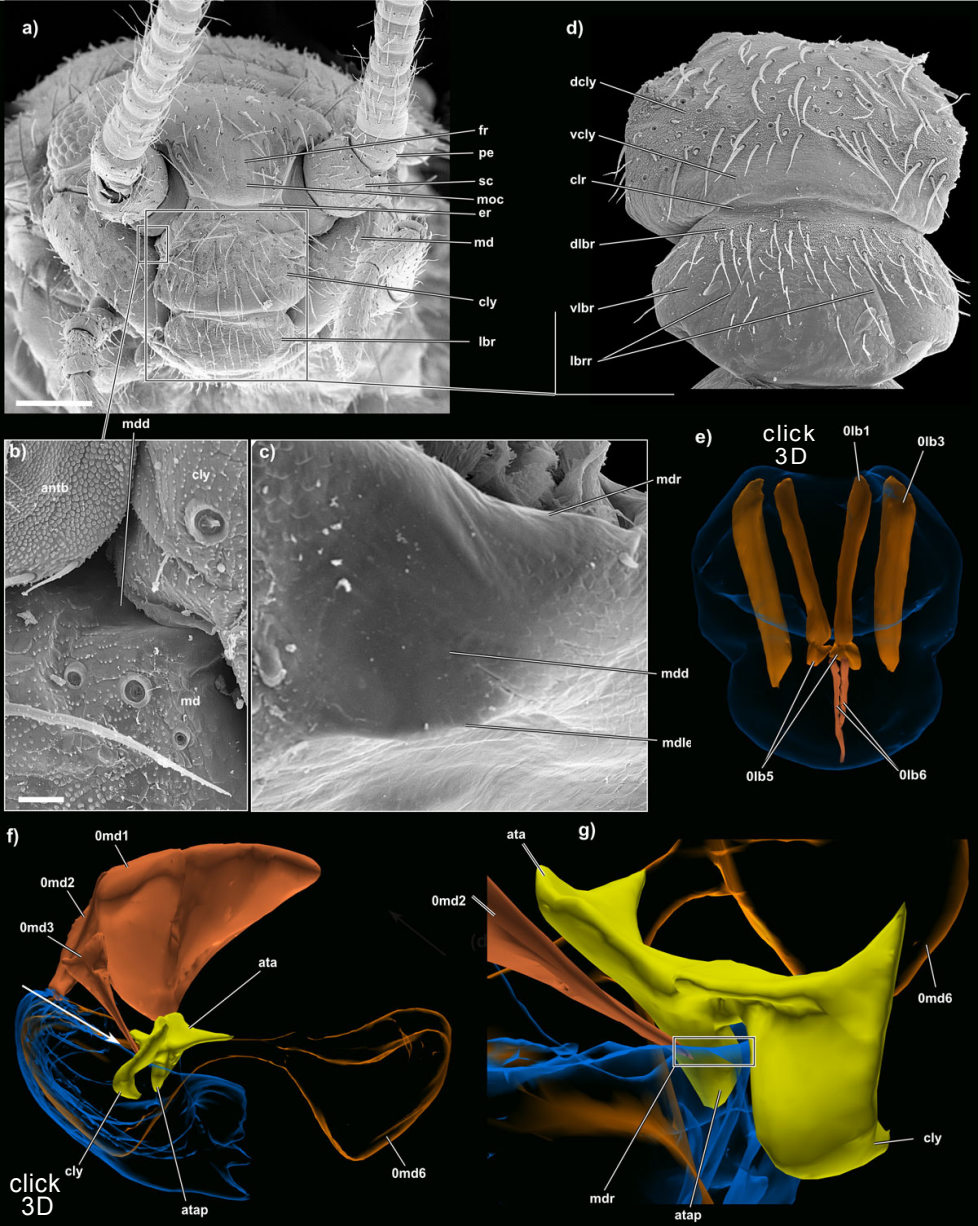
**SEM micrographs and 3D reconstructions of *Tricholepidion gertschi*;**. **a) **frontal overview of the head; scale bar 200 μm; **b) **detail of the externally visible part of the anterior mandibular articulation; scale bar 20 μm; **c) **detail of the anterior mandibular articulation complex. **d) **detail of the clypeal region and the labrum; **e) **extrinsic and intrinsic labral musculature; **f) **frontal view of the anterior mandibular articulation complex illustrating the caliper-like form built by the anterior tentorial arm and the clypeus. The white arrow shows the viewing angle for Figure 5g); **g) **dorsolateral view of the anterior mandibular articulation complex. Abbreviations: antb, antennal base; ata, anterior tentorial arm; atap, anterior tentorial apodeme; clr, clypeal ridge; cly, clypeus; dcly, dorsal clypeal area; dlbr, dorsal labral area; er, epistomal ridge; fr, frons; lbr, labrum; lbrr, intralabral ridges; md, mandible; mdd, mandibular depression; mdle, mandibular lateral edge; mdr, mandibular ridge; moc, middle occelus; pe, pedicellus; sc, scapus; vcly, ventral clypeal area; vlbr, ventral labral area. Please click on the figure to activate the 3D content. For muscle references see main text. Images not to scale to each other.

In anterior view the undivided clypeus is formed like a dorsoventrally elongated hexagon (Figure 5a). The well-developed epistomal ridge forms its medio-dorsal and the antennal bases its dorso-lateral delimitation. The clypeus is covered by the same two sensillum types like the rest of the head capsule except for its ventral part near the clypeo-labral ridge where it is smooth and entirely devoid of setae (Figure 5d).

The epistomal ridge is located at half height of the antennal bases. An interantennal ridge is absent. The frons is roundish in lateral view (Figure 4b). It continues ventrally into the genal area which also harbours the posterior mandible articulation (Figure 4b). In dorsal view the frons appears like a semi-circle, its corners almost meeting the eyes anteriorly (Figure 3a). The frontal sutures and the coronal suture form an inverted Y when seen from dorso-frontal. The frontal sutures are barely visible, the coronal suture continues from the frontal sutures posteriorly until the postocciput (Figure 3a).

The head-wise part of the posterior mandibular articulation comprises a depression and a pyramidal condylus with its tip oriented ventrally (Figure 4a). The condylus formed at the posterior end of the mandible lies in the depression so that a ginglymus is formed. The straight occipital ridge (in dorsal view; Figure 3a) clearly separates the frons from the rectangular occiput. The width of the occiput (in lateral view) corresponds to the width of the compound eyes. The occiput is posteriorly delimited by a strong postoccipital ridge. The postoccipital ridge is equally well developed and serves as an attachment point for several thoracic muscles. The postocciput is one third the length of the occiput (in dorsal view; Figure 3a) and wider than the rest of the head capsule. In the lateral region the postocciput bears three very long (~150 μm) trichoid sensilla which are laterally oriented. For a different interpretation of the above mentioned head regions here considered as occiput and postocciput see the discussion.

### Cephalic endoskeleton

**Figure 6:**
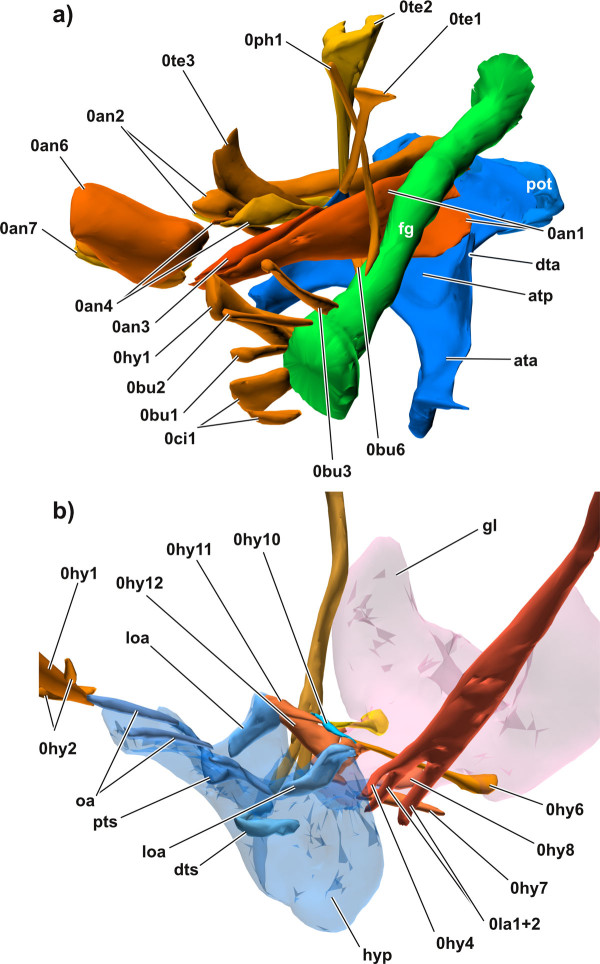
**3D reconstructions in dorsolateral view**. **a) **the musculature of the antenna, cephalic digestive tract, and tentorium; **b) **hypopharynx and efferent duct of salivary glands with the corresponding musculature. Abbreviations: ata, anterior tentorial arm; atp, anterior tentorial plate; dta, dorsal tentorial arm; dts, distal transverse sclerite; fg, foregut; gl, efferent duct of salivary glands; loa, loral arm; oa, oral arm; pot, posterior tentorium; pts, proximal transverse sclerite. Please click on the figure to activate the 3D content. For muscle references see main text.

The cuticular endoskeleton (Figures 3b, 4c, 6a) is composed of two main elements: a posterior tentorium and an anterior tentorium. The anterior tentorium is composed of paired anterior and dorsal tentorial arms and an anterior tentorial plate. The posterior tentorium is connected to the anterior tentorial plate by small muscles (0te5 + 6). The anterior tentorial pits are externally not visible. They are located ventral to the voluminous antennal bases in a cavity delimited by the mandibles, the clypeus and the antennae (Figure 5b). The massive, anterior tentorial arms emerge from the anterior tentorial pits and coalesce into the anterior tentorial plate at the level of the transverse mandibular tendon. At the point of fusion the dorsal tentorial arms emerge. They are not in contact with the head capsule but suspended to it by two muscle bundles (0te2 and 0te4). Posterior to the dorsal arms the anterior plate narrows and widens again at level of the posterior mandibular articulation, just where the posterior tentorium begins.

Musculature: M. tentoriofrontalis posterior (**0te1**): insertion directly anterior of 0md1 (muscle "spt" of Chaudonneret [33]); O, at the dorsal tentorial arms I, frons, near the occipital ridge anterior of the 0md1. M. tentoriofrontalis anterior (**0te2**): the insertion in between the muscle bundles of 0md1 supports homologisation with the muscle "spot" of Chaudonneret [33]; O, at the dorsal tentorial arms together with 0te1; I, frons. M. tentoriofrontalis dorsalis (**0te3**): O, anterior side of the dorsal tentorial arm; I, frons near the dorsal part of the antennal base. M. posterotentorialis (**0te4**): absent. M. tentoriotentorialis longis (**0te5**): O, ventrolaterad on the anterior corpotentorium; I, mesad on the posterior tentorium. M. tentoriotentorialis brevis (**0te6**): O, anterior tentorium, along the gap towards the posterior tentorium; I, posterior tentorium, along the gap towards the anterior tentorium.

### Labrum

The convex labrum (Figures 5d + e) is covered with a stripe of trichoid sensilla and small sculptures of the exoskeleton immediately below the clypeolabral ridge. On the remaining external labral surface trichoid sensilla occur sporadically. The labrum partly covers the mandibles in frontal view and is moveably connected to the clypeus. Two thin dorsoventral ridges occur on the frontal side of the labrum but do not reach its apex.

Musculature: M. frontolabralis (**0lb1**): O, mesally on the epistomal ridge; I, mesally on the inner basal wall of the labrum. M. frontoepipharyngalis (**0lb2**): absent. M. epistoepipharyngealis (**0lb3**): this muscle can easily be confused with the 0lb2. The origin of 0lb3 lies clearly only on the epistomal ridge. O, laterally on the epistomal ridge; I, basal epipharyngeal wall. M. labralis transversalis (**0lb4**): absent. M. labroepipharyngealis (**0lb5**): O, basal labral wall; I, basal wall of epipharynx. M. labrolabralis (**0lb6**): O, mesobasal labral wall in between the two bundles of 0lb5; I, medioapical area of labrum.

### Antennae

The antennal foramina are directed fronto-laterad. The membranous antennal bases are half as long as the scapus (Figure 5a). The scapus is approximately one third longer and wider as the pedicellus. The antennomeres of the flagellum become gradually shorter from the base towards approximately half of the flagellum and subsequently gradually elongate again. At the distal region each flagellomere is longer than the basal flagellomere and divided into two subarticles (Figure 4b). Overall the flagellum becomes thinner from base to tip.

Musculature: M. tentorioscapalis anterior (**0an1**): O, mesally on the corpotentorium; I, anterior margin of the scapus. M. tentorioscapalis posterior (**0an2**): O, in the posterior part of the corpotentorium; I, posterior basal margin of the scape. M. tentorioscapalis lateralis (**0an3**): O, at the anterior base of the dorsal tentorial arms; I, dorsal basal margin of the scape. M. tentorioscapalis medialis (**0an4**): O, at the dorsal tentorial arms directly over 0te1; I, dorsal basal margin of the scape. M. frontopedicellaris (**0an5**): absent. M. scapopedicellaris lateralis (**0an6**): O, broadly on the dorsolateral base of the scape; I, dorsolateral base of the pedicellus. M. scapopedicellaris medialis (**0an7**): O, ventrolateral base of the scape; I, ventrolateral base of the pedicel. M. intraflagellaris (**0an8**): absent. M. interampularis (**0ah1**): absent. M. ampulloaortica (**0ah2**): absent. M. ampullopharyngealis (**0ah3**): absent. M. ampullofrontalis (**0ah4**): absent. M. frontofrontalis (**0ah5**): absent.

### Mandibles

**Figure 7:**
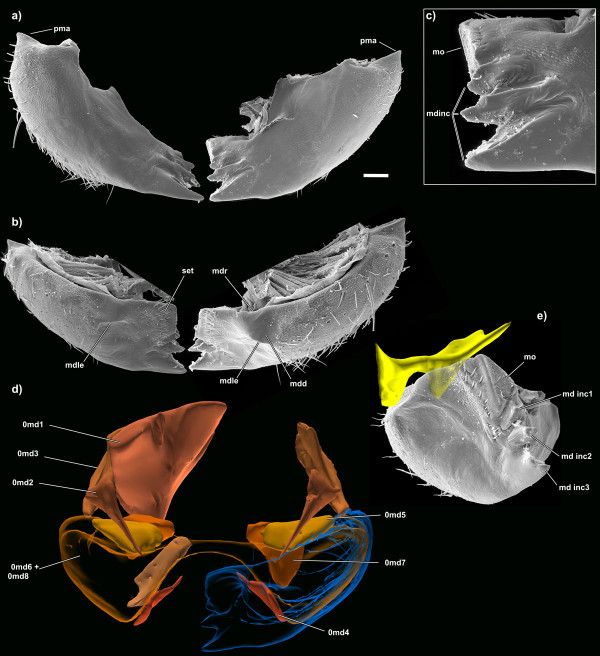
**SEM micrographs and 3D reconstructions of the mandibles of *Tricholepidion gertschi*;**. **a**) posterior view; **b) **anterior view; **c) **detail of the gnathal area of the left mandible; **d) **mandibular musculature; **e) **mesal view. The yellow 3D reconstruction again shows the spatial relation of the mandible and the head part of the anterior mandibular articulation complex. The part lying within the mandible is semitransparent. Abbreviations: md inc 1-3, mandibular incisivi; mdd, mandibular depression; mdle, mandibular lateral edge; mdr, mandibular ridge; mo, mola; pma, posterior mandibular articulation; set, setae. Images not to scale to each other, scale bar for a) and b) 100 μm. Please click on the figure to activate the 3D content. For muscle references see main text.

**Figure 8:**
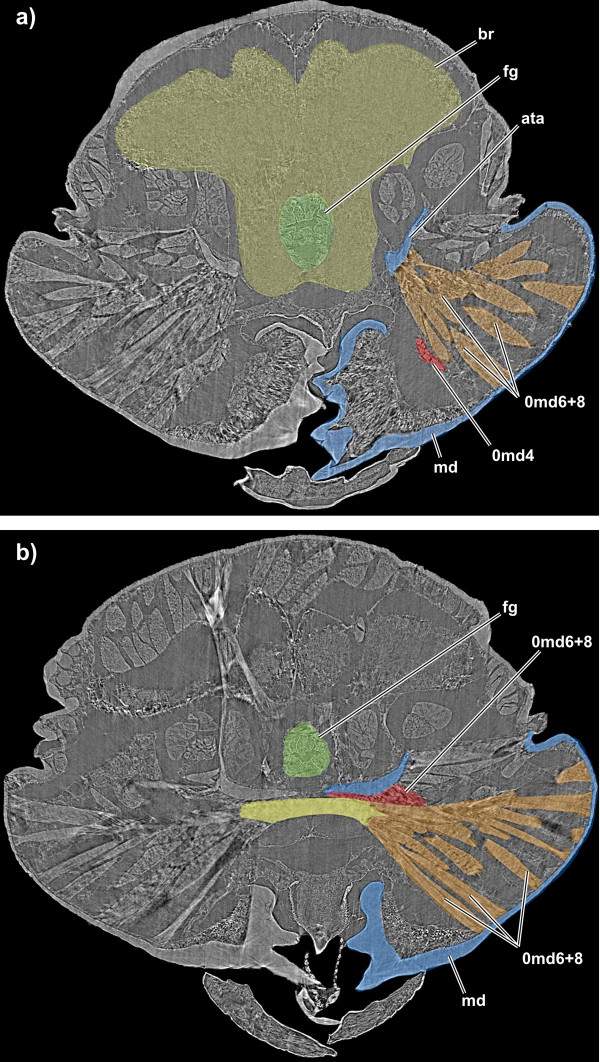
**Frontal virtual SR-microCT sections through the head of *Tricholepidion gertschi *with **a) **the 0md4 and 0md8 muscles coloured on the right side of the animal to show the presence of the 0md4 and the insertion point of the 0md8, and with **b) **the ligamentous connection of 0md6 between both mandibles in yellow color and the muscular connection to the anterior tentorium in red colour**. Abbreviations: ata, anterior tentorial arm; br, brain; fg, foregut; md, mandible. For muscle references see main text.

The mandibles (Figure 7) are formed like an elongated bowl in dorsal view with an oval dorso-mesally oriented opening to which several muscles attach (Figures 5f, 7d, 8). The mandibles are overall rigidly sclerotised with the greatest wall thickness at the gnathal region (incisivi and mola) and the posterior and anterior mandibular articulation regions (Figures 7c + e). Anteriorly, the mandibles form a sharp median edge with a dorsal mola and three ventral incisivi (Figures 7c + e). The gnathal edges are almost symmetrical on both mandibles.

The posteriormost part of the mandibles bears the posterior mandibular articulation which is continuous with the overall form of the mandible (Figure 7a + b), so that no distinct knob is formed. The loose anterior mandibular articulation complex is situated at height of the dorsal part of the mola, a short distance distal to it. It is composed of two parts: the headwise part is a "caliper-like" structure formed by parts of the anterior tentorial arms and the clypeus (Figure 5f + g). The mandibular part of the articulation consists of a thickened mandibular dorsal margin (the mandibular ridge), a depression ventral of this ridge, and a lateral edge delimiting the depression laterally (Figure 5c).

The caliper is "wrapped" around the mandibular edge, its clypeal part forms a process which touches the mandibular depression and fits into it. The posterior part of the caliper (a ventral apodeme of the anterior tentorial arm) reaches into the lumen of the mandible directly posterad the mandibular ridge (Figure 5g). The two processes (clypeal and tentorial) of the caliper thus prevent an antero-posterad movement of the mandible.

The mola is almost formed like a right-angled triangle in lateral view (Figure 7e), with the hypotenuse directed anteriorly. The anterior edge of the mola is armed with a row of setae which are oriented medially towards the chewing surface of the mola. The surface of the mandibles is covered with trichoid sensilla on parts of the anterior side only. From the anterior mandibular articulation towards the incisivi as well as on the whole posterior side, the mandibles are devoid of sensilla.

Musculature: M. craniomandibularis internus (**0md1**): O, dorsal parts of the head, anterior of the postoccipital ridge and on this ridge; one muscle bundle also posterior of the postoccipital ridge; I, tendon originating from the proximal part of the posterior mandibular edge. M. craniomandibularis externus anterior (**0md2**): O, gena, anterior of the compound eye; I, tendon originating from the proximal part of the anterior mandibular edge, directly lateral to the anterior articulation complex. M. craniomandibularis externus posterior (**0md3**): O, directly posterior of the compound eye, with several bundles also at the postoccipital ridge; I, tendon originating from the anterior mandibular edge near the posterior articulation. M. hypopharyngomandibularis (**0md4**): O, small sclerite close to the loral arm of the hypopharynx; I, proximal inner side of the anterior wall of the mandible. M. tentoriomandibularis lateralis superior (**0md5**): O, ventrally at the anterior tentorial arm; I, anterior mandibular rim between 0md2 and 0md3. M. tentoriomandibularis lateralis inferior (**0md6**): O, the whole inner wall of the mandible except for the region near the incisivi and mola; I, in the same region of the other mandible and with a few muscle bundles also at the ventral anterior area of the corpotentorium. M. tentoriomandibularis medialis superior (**0md7**): O, at the ventral base of the anterior tentorial arms; I, proximal of the posterior articulation at the posterior mandibular rim. M. tentoriomandibularis medialis inferior (**0md8**): O, at the transition of the corpotentorium and the anterior tentorial arm below 0md7; I, mediodorsal wall of the mandibular cavity.

### Maxillae

**Figure 9:**
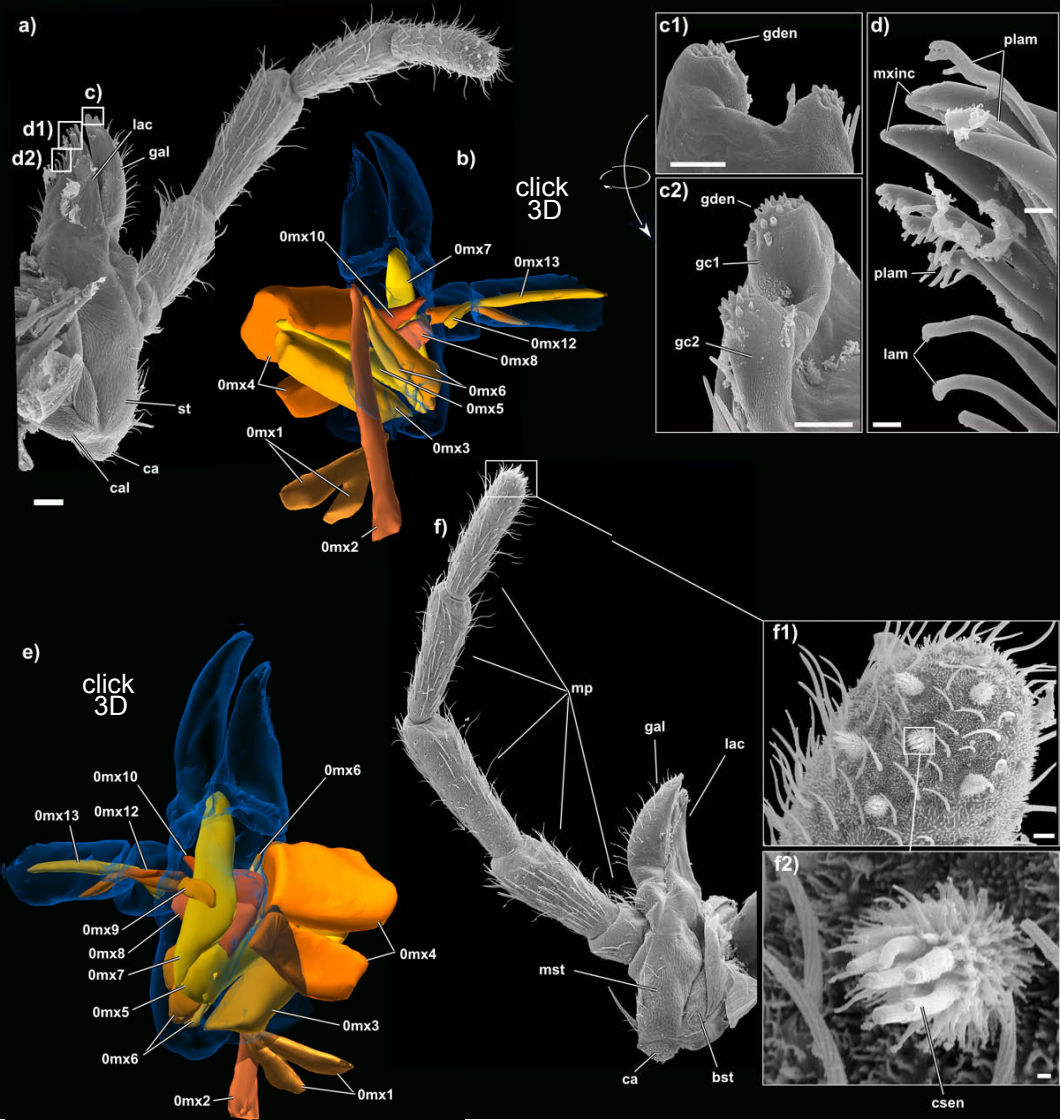
**SEM micrographs and 3D reconstructions of the maxillae of *Tricholepidion gertschi***. **a) **posterior view of the outer anatomy; **b) **the muscle equipment in posterior view; **c1) **detail of the apical area of the galea (posterior view); **c2) **detail of the apical area of the galea (meso-anterad view); **d) **detail of the apical and subapical area of the lacinia; **e) **the muscle equipment in anterior view; **f) **anterior view of the outer anatomy; **f1) **detail of the apical area of the maxillary palpus; **f2) **detail of a sensillum at the apex of the maxillary palpus. Abbreviations: bst, basistipes; ca, cardo; cal, lever of the cardo; csen, conical sensillum; gal, galea; gc1 + 2, first and second apical cones of galea; gden, galeal denticles; lac, lacinia; lam, lamellae; mp, maxillar palpus; mst, mediostipes; mxinc, maxillary incisivi; plam, pectinate lamellae; st, stipes. Images not to scale to each other, scale bar for **a) **and **f) **100 μm; for **c) **+ **d) **+ f1) 10 μm; for f2) 1 μm. Please click on the figure to activate the 3D content. For muscle references see main text.

The body of the maxillae (from cardo to galea) is three times longer than wide in overall shape (Figure 9a). The cardo is approximately triangular and contains a medially oriented lever (Figure 9a) serving as attachment for the M. craniocardinalis (0mx1). The cardo bears some setae and is moveably connected to the stipes by a very narrow articular membrane lying at the base of the cardo-stipital ridge.

The stipes is composed of a narrow basistipes and a much larger mediostipes bearing the palpus, galea and lacinia (Figure 9f). The whole stipes is devoid of sensilla except for the part directly posterior to the palpal base (the area which is externally visible in lateral view; see Figure 4). The base of the maxillary palpus is surrounded by protrusions of the stipes forming a ring around the palpal foramen (palpifer).

The palpus is five-segmented and densely covered with trichoid sensilla. The first segment is one third as long as the second one. The third one is slightly longer than the second one, the fourth and fifth are as long as the second one. Each segment is slightly thinner than the preceding one. The fifth segment bears distally six special sensilla formed by a basal cylindrical segment densely covered with microtrichia and four to six tubular extensions on the tip of the cylindrical base (Figures 9f1 + f2). These sensilla are arranged in a pentagon with the 6th sensillum at its center.

The galea is sickle-shaped (Figure 9a + f), distally covered with setae and bears apically two sensorial projections with several minute cones at their apices, each equipped with a terminal pore (Figures 9c1 + 2). The lacinia is also sickle-shaped (Figure 9a + f); it bears three apical, mesally oriented incisivi with a single apically fringed lamella in between (Figure 9d1), three subapical lamellae with fringed apices (pectinate lamellae, Figure 9d1 + d2), and further basally one row of six entire lamellae (partly shown in Figure 9d2). A row of trichoid sensilla follows directly posterior to the setae from half the length of the lacinia to the base of the lacinia (Figure 9a). Dentisetae (as characteristic for Palaeoptera [32]) are absent.

Musculature: M. craniocardinalis (**0m**x**1**): O, laterally at the postoocipital ridge; I, basal cardinal process. M. craniolacinialis (**0m**x**2**): O, distal of 0mx1 at the postoocipital ridge; I, cuticular tendon at the basal edge of lacinia together with 0mx6. M. tentoriocardinalis (**0m**x**3**): O, at the lateral part of the posterior tentorium; I, inner wall of the cardo. M. tentoriostipitalis anterior (**0m**x**4**): O, ventral on the corpotentorium; I, at the posterior stipital rim. M. tentoriostipitalis posterior (**0m**x**5**): O, lateral on the corpotentorium; I, at the basal inner wall of the stipes. M. stipitolacinialis (**0m**x**6**): O, posterior wall of the stipes, basal to the cardostipital ridge; I, basal proximal edge of lacinia, at a common cuticular tendon with 0mx2. M. stipitogalealis (**0m**x**7**): O, posterior wall of the stipes, basal to the cardostipital ridge next to 0mx6; I, basal edge of galea. M. stipitopalpalis externus (**0m**x**8**): O, posterior wall of the stipes, opposite of the palpus; I, posterior rim of the first palpomere of the maxillary palpus. M. stipitopalpalis medialis (**0m**x**9**): O, medially at the posterior wall of the stipes; I, ventral edge of palpomere 1. M. stipitopalpalis internus (**0m**x**10**): O, posterior inner wall of the stipes, opposite of the palpus; I, anterior rim of the first palpomere of the maxillary palpus. M. stipitalis transversalis (**0m**x**11**): O, outer stipital wall, near the palpus; I, inner stipital wall, near the palpus. M. palpopalpalis maxillae primus (**0m**x**12**): O, basal edge of palpomere 1; I, basal edge of palpomere 2. M. palpopalpalis maxillae secundus (**0m**x**13**): O, mesal edge of palpomere 2; I, mesal edge of palpomere 3. M. palpopalpalis maxillae tertius (**0m**x**14**): O, basal edge of palpomere 3; I, basal edge of palpomere 4; M. palpopalpalis maxillae quartus (**0m**x**15**): unclear.

### Labium

The labium (Figure 2a) is divided into postmentum, prementum, glossae, paraglossae and palpus. All externally visible parts are covered with trichoid sensilla in the same density as the rest of the head capsule. The postmentum is an almost rectangular plate. The prementum is also rectangular in ventral view and bears a deep median premental cleft. Glossae and paraglossae are short, the former finger-like and a bit longer than the broadened paraglossae. The palpi are four-segmented; a short basal segment is followed by two elongate segments and a widened apical segment. The anterior (or ventral) surface of the apical segment shows a median cleft and is densely covered with trichoid sensilla. Two additional sensilla types are present in the distal region. Towards the apical margin there are three brush-like clusters composed of thin tubular extensions with flattened tips (Figures 2b + d). Mesally three wart-like groups of sensilla occur that closely resemble those on the apical segment of the maxillary palpus; each sensillum is composed of a basal cylindrical segment densely covered by microtrichia and a single distal tubular extension (Figures 2b + c).

Musculature: M. postoccipitoglossalis medialis (**0la1**): O, posterior side of the postoccipital ridge; I, proximal area of the glossal base. M. postoccipitoglossalis lateralis (**0la2**): O, posterior side of the postoccipital ridge, right next to 0la1; I, dorsolateral area of the glossal base. M. postoccipitoparaglossalis (**0la3**): O, posterior side of the postoccipital ridge, lateral of 0la1 & 0la2; I, distal base of the paraglossa. M. postoccipitopraementalis (**0la4**): O, posterior side of the postoccipital ridge, right next to 0la1 & 0la2; I, inner proximal wall of the prementum. M. tentoriopraementalis (**0la5**): O, lateral area of the posterior tentorium; I, laterobasal edge of prementum. M. tentorioparaglossalis (**0la6**): O, lateral area of the posterior tentorium, right next to 0la5; I, paraglossa, close to the labial palpus. M. tentorioglandularis (**0la7**): O, lateral posterior area of the corpotentorium; I, labial gland. M. submentopraementalis (**0la8**): O, medially on the postmentum; I, mediobasal edge of prementum. M. postmentomembranus (**0la9**): unclear. M. submentomentalis (**0la10**): absent. M. praementoparaglossalis (**0la11**): O, distal on the basal edge of the prementum; I, basal edge of paraglossa. M. praementoglossalis (**0la12**): O, more mesally on the basal edge of the prementum, right next to 0la11; I, basal edge of glossa. M. praementopalpalis internus (**0la13**): O, mesally on the prementum; I, anterior basal edge of labial segment 1. M. praementopalpalis externus (**0la14**): O, mesally on the prementum, right next to 0la13; I, posterior basal edge of labial segment 1. M. praementomembranus (**0la15**): O, anterolateral area of the postmentum; I, anteromedial area of postmentum. M. palpopalpalis labii primus (**0la16**): O, mediobasal edge of labial palpomere 1; I, medial and distal edge of palpomere 2. M. palpopalpalis labii secundus (**0la17**): O, mediobasal edge of palpomere 2; I, basal edge of the palpomere 3.

### Hypopharynx, epipharynx, and salivarium

The hypopharynx has a wide lumen and is strengthened by a suspensorium made of several sclerites (Figure 6b) to which muscle bundles attach (see below). Two transverse sclerotized ribbons are present in the anterior part of the hypopharynx and cross the anterior surface. The proximal transverse sclerite (pts; Figure 6b) originates from the oral arm (oa), the distal transverse sclerite (dts, Figure 6b) originates from the distal part of the suspensorium below the loral arm (loa). The epipharynx (= inner side of the clypeolabrum) is a concave structure and bears two fields of hairs on the inner side. The mandibles fit into the concave space of the epipharynx. When closed, the right mandible is positioned a short distance in front and more ventrally of the left one. The joint, unpaired efferent duct of the salivary glands and labial nephridia is bowl-shaped (Figure 6b) and opens into the salivarium directly posterior of the hypopharynx.

Musculature: M. frontooralis (**0hy1**): O, frons, near the antennal base; I, oral arms of the suspensorial sclerites. M. tentoriooralis (**0hy2**): O, anterior tentorial arm, near the anterior tentorial pit; I, one muscle bundle on the oral arms of the suspensorial sclerites, one bundle at the lateral buccal wall: both bundles are well separated from 0hy1. M. craniohypopharyngealis (**0hy3**): O, posterior tentorial arms; I, suprasalivarial sclerite. M. postoccipitalohypopharyngealis (**0hy4**): O, posterior wall of the postoccipital ridge, together with 0la1 & 0la2; I, hypopharyngeal fulcrum. M. tentoriosuspensorialis (**0hy5**): O, anterior margin of the posterior tentorium; I, suspensorium of the hypopharynx. M. postmentoloralis (**0hy6**): O, anterior part of the postmentum; I, loral arm of the hypopharyngeal suspensorium. M. praementosalivaris anterior (**0hy7**): O, distolateral wall of the prementum, close to the labial palpus; I, lateral wall of salivarium. M. praementosalivaris posterior (**0hy8**): O, medially on the basal part of the prementum; I, posterior wall of salivarium. M. oralis transversalis (**0hy9**): unclear. M. loroloralis (**0hy10**): O, loral arm of suspensorial sclerite; I, loral arms of the suspensorial sclerites on the other side. M. lorosalivarialis (**0hy11**): O, hypopharyngeal suspensorium; I, loral arm of the hypopharynx. M. hypopharyngosalivaris (**0hy12**): O, loral arm of the hypopharynx, right next to 0hy11; I, salivarial orifice. M. anularis salivarii (**0hy13**): unclear.

### Foregut

The foregut has a wide lumen and is not distinctly subdivided into pharynx and oesophagus. Various muscles hold the foregut into position (0ci1, 0bu1-3, 0bu5 + 6, Figure 6a).

Musculature: M. clypeopalatalis (**0ci1**): two distinct muscle bundles. O, postclypeus; I, roof of the cibarium. M. clypeobuccalis (**0bu1**): O, clypeus, near the epistomal ridge; I, roof of the bucca. M. frontobuccalis anterior (**0bu2**): O, frons dorsal of the epistomal ridge; I, dorsal buccal wall. M. frontobuccalis posterior (**0bu3**): O, more posterior than 0bu2 on the frons; I, dorsal buccal wall, posterior of 0bu2. M. tentoriobuccalis lateralis (**0bu4**): absent. M. tentoriobuccalis anterior (**0bu5**): O, anteriormost part of the corpotentorium between the anterior tentorial arms; I, ventral wall of the bucca, directly behind the anatomical mouth. M. tentoriobuccalis posterior (**0bu6**): O, laterally on the dorsal wall of the corpotentorium; I, lateral wall of the foregut at height of the 0ph1. M. verticopharyngealis (**0ph1**): O, frons right next to the 0te4; I, dorsal wall of the foregut, posterior to the supraoesophagial ganglion. M. tentoriopharyngealis (**0ph2**): O, laterally on the dorsal wall of the corpotentorium right next to 0bu6; I, ventral wall of the foregut, beneath 0ph1. M. postoccipitopharyngealis (**0ph3**): absent. M. anularis stomodaei (**0st1**): ring muscle layer that covers the entire foregut. M. longitudinalis stomodaei (**0st2**): longitudinal muscle layer covering the entire foregut, right next to 0st1.

## Discussion

In this study we characterize all muscles and endoskeletal features of the head of *T. gertschi*. The description of the outer anatomy largely conforms with the one of Wygodzinski [10]. In contrast to Wygodzinski [10] we interpret the general head organisation as orthognathous (only the labial palpi point to the rear (Figures 2a & 4b)), although we believe that this incongruence is due to the different use of the terms "orthognathous" and "hypognathous". In the Anglo-Saxon language the terms "hypognathous" and "orthognathous" are often used synonymously [34]. The interpretation of the region dorsal of the eyes as occiput (Figures 3a, 4b) - and consequently the designation of the postoccipital area - is unclear. Matsuda [35] and Snodgrass [36] proposed a tripartite gnathocephalon where the postocciput is regarded as the labial segment and the occiput as the maxillary segment. They are supposed to be separated by the occipital ridge and the postoccipital ridge. Accordingly, the frons harbours the eyes. However, we consider cephalic ridges and sulci as lines of mechanical strengthening (ridges) or weakening (sulci) which either deflect mechanical strain or serve as predetermined breaking points during ecdysis. Thus, they are not associated with any head segments [37-40] or head regions such as occiput, postocciput, or frons. For the time being we adhere to the latest accounts on this problem [11,14], which favour the interpretation of the ridge posterior of the eyes as the postoccipital ridge.

In general, the cephalic morphology of *T. gertschi *is characterized by the presence of several potential autapomorphies: absence of 0lb2 and 0hy10, apex of the labial palpi with two different types of sensilla, and a clypeus and labrum each with two distinguishable subareas. Due to the absence of an intraclypeal ridge we refrain from interpreting the clypeus as distinctly separated into ante- and postclypeus despite the differing surface structure (Figure 5d).

### Monophyletic Dicondylia and Pterygota are corroborated

Generally, the monophyly of Dicondylia and Pterygota is well supported by molecular and morphological data [2,41] even though some authors doubted this view [42,43]. Our phylogenetic analysis including *T. gertschi *as well as *L. saccharina, T. domestica *and *A. formicaria *corroborated the monophyly of Dicondylia. Potential autapomorphies are the presence of a coronal suture, cuticular dorsal tentorial arms, presence of an additional anterior mandibular joint (for which the group is named), presence of M. labroepipharyngealis (0lb5), M. verticopharyngealis (0ph1), M. tentoriopharyngealis (0ph2) and the five-segmented maxillary palpus.

**Figure 10:**
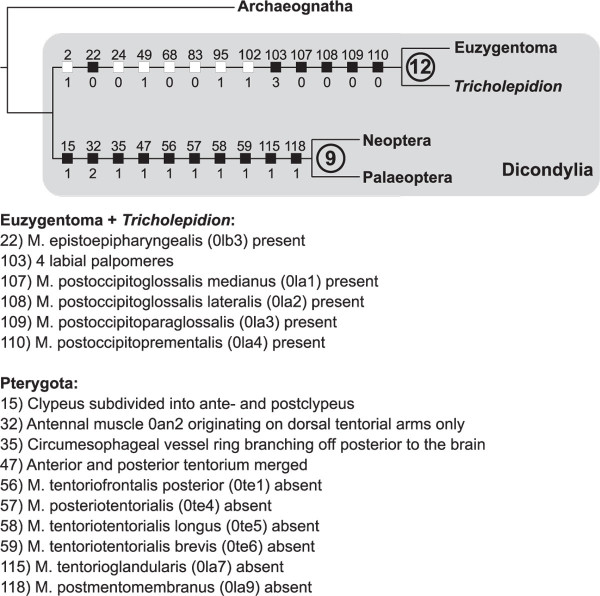
**Strict consensus, focused on the phylogenetic position of *T. gertschi*, of the two equally parsimonious trees derived from the TNT analysis of the morphological character matrix**. Bremer support values are given in bold circled numbers. Unambiguous, non-homoplastic character optimizations are detailed below the tree in an abbreviated version. Non-homoplastic character changes are indicated with black squares, homoplastic characters with white squares. Trait numbers are indicated above squares, state changes below. For muscle references see main text. See additional file 6 for a complete tree with all pterygote taxa.

The monophyly of winged insects is strongly supported (BR 9; Figure 10). Unambiguous autapomorphies of Pterygota are the divided clypeus (15:1), the origin of the antennal muscle 0an2 at the dorsal tentorial arms (32:2; although character states are shifting among Neoptera), the fusion of the pre- and posttentoria (47:1) and the loss of several tentorial muscles (56-59:1), as well as the absence of a circumesophageal vessel ring (35:1) and the loss of labial musculature (0la7; 115:1 & 0la9; 118:1). The loss of hypopharyngeal muscles 0hy6 (131:1) and 0hy11 (133:1) may represent further autapomorphies of Pterygota, but the ancestral states of these characters remain ambiguous due to the lack of a more distantly related outgroup.

### Head data supports monophyletic Zygentoma

The position of *T. gertschi *was discussed controversially: It was considered as sistergroup to Euzygentoma [17,18], as sistergroup to Euzygentoma + Pterygota [15,16], or as sistergroup to Nicoletiidae and Ateluridae [10,19] within Euzygentoma. Analysis of cephalic data did not support a hypothesis about the phylogenetic position of *T. gertschi*, since the M. hypopharyngo-mandibularis (0md4) was considered absent [11,14] and due to the existence of a ligamentous connection of M. tentoriomandibularis lateralis inferior (0md6) between the mandibles [11,44,45]. In our specimens a M. hypopharyngo-mandibularis (0md4) is clearly visible, origin, insertion, and course of this muscle are in line with its organisation in other dicondylian taxa. Thus, only the ligamentous connection of muscle M. tentoriomandibularis lateralis inferior (0md6) between the mandibles [11,44,45], which is shared with Archaeognatha, is left as an argument against the monophyly of Zygentoma. It has to be emphasized that parts of this muscle are also in contact with the anterior tentorium despite the presence of a ligamentous connection. Archaeognatha possess only a ligamentous connection and the remaining insects (excl. *Tricholepidion*) exhibit an origin of this muscle exclusively on the tentorium. Our phylogenetic analysis corroborates monophyletic Zygentoma despite the inclusion of the mandible ligament in the character matrix (character 69) with high support (BR 12; Figure 10). Synapomorphies of Euzygentoma and *T. gertschi *revealed in our study concern the composition of the labial musculature (107:0; 108:0; 109:0; 110:0). Euzygentoma and *T. gertschi *possess a remarkable set of extrinsic labial muscles originating from the postoccipital region and extending dorso-ventrally through the whole head into the labium. Archaeognatha and Pterygota clearly do not possess this set of labial muscles [32,44,46]. The situation in Protura, Collembola, and Diplura is unclear due to homologisation problems of the labial structures and the corresponding muscles. Both, T. *gertschi *and Euzygentoma, possess four labial palpomeres (103:3). Also, the intralabral muscle equipment is characterized by an additional muscle, the M. epistoepipharyngalis (0lb3; 22:0). In the present analysis the low number of ommatidia (less than 80; 2:1) is a homoplastic character since Grylloblattodea also show reduced eyes [30] which however may be due to convergence. Wing-like tentorial processes reaching into the lumen of the mandible are also present in Odonata (49:1; [32]). A M. labrolabralis (0lb6; 24:0) and the cylinder shaped posterior mandibular joint (68:0) are characters also present in Ephemeroptera [14], the number of lacinial incisivi (83:0) is shared with the grylloblattodean *Galloisiana yuasai *[30]. Except for the (homoplastic) loss of the mandible ligament (see above), the loss of ocelli (but see below), and the loss of M. verticopharyngealis (0ph1) we found no cephalic apomorphies characterizing Euzygentoma. Evidence from cephalic characters therefore suggests monophyletic Zygentoma, with *T. gertschi *as sister to Euzygentoma.

### Other character systems

With few exceptions most molecular studies advocate monophyletic Zygentoma. Based on secondary structure alignment of the 18S unit Kjer [5] recovered *Tricholepidion *as sistergroup to Odonata with low support while Giribet [47], using a more inclusive molecular dataset and a compilation of published morphological data, proposed *Tricholepidion *as sister taxon to Euzygentoma + Pterygota with low support. All other molecular works supported monophyletic Zygentoma [3,4,6,7].

The monophyly of Zygentoma was further argued by the occurrence of sperm conjugation (or sperm pairing) in *T. gertschi *and Lepismatidae [48]. Zrzavý [1] considered this a weak argument, since the mode of sperm aggregation seems to be quite diverse among Zygentoma (reviewed by Dallai et al. [19]). In a detailed study on the sperm ultrastructure and sperm pairing mode of *T. gertschi *Dallai et al. [20,21] discovered that the spermatozoa morphology in *T. gertschi *resembles the ancestral state of insects with a 9 + 9 + 2 axonemal pattern and accessory tubules with 16 protofilaments. The sperm pairing in *T. gertschi *is a true fusion between two spermatozoa along the entire sperm head region, which is different from sperm aggregations in other Zygentoma [19]. Characters related to sperm conjugation accordingly provide no convincing evidence in favour or against zygentoman paraphyly. The same concerns the number of ovarioles that seems to be plesiomorphic in *T. gertschi *(seven, as in Archaeognatha [10]). The ovariole number is reduced in euzygentomans (five in Lepismatidae; three in Nicoletiidae; [49]) and highly variable in Pterygota [49].

Other possible synapomorphies of Euzygentoma and *T. gertschi *include similarities in the mating behaviour of *T. gertschi *and Lepismatidae [50], a unique type of sensillum on the terminal filament of males of *T. gertschi *and some Nicoletiidae [10], and the widened apical segment of the labial palpus. Although the similarity of the main elements of mating is remarkable, the mating behaviour among Zygentoma is variable and especially difficult to compare between Archaeognatha and Zygentoma: Archaeognatha show at least three distinctly different modes of sperm transfer, another two are hypothesized for *Petrobiellus *and *Mesomachillis *species [46]. The lack of a robust phylogeny of Archaeognatha presently impedes a polarization of the modes of sperm transfer in this taxon. The scattered occurrence of special sensilla on the terminal filaments of males of some Nicoletiidae (including some Ateluridae) likewise demands a robust hypothesis on the interrelationships among blind silverfish to clarify the phylogenetic significance of the presence of these sensilla in *T. gertschi*. The widening of the apical labial palpus segment is paralleled in males of several Machilidae and Meinertellidae (e.g. *Silvestrichilis*, *Trigoniophthalmus*, *Promesomachilis*, nearly all Meinertellidae [46]) and, hence, seems to be homoplastic.

Also, we interpret the five-segmented tarsi [10] not as an autapomorphy of *T. gertschi*, since these occur as well in several pterygote taxa and the phylogenetic significance is therefore unclear. Zrzavý [1] considered five-segmented tarsi as the plesiomorphic condition for Pterygota. Taking into account the tarsus configuration in *T. gertschi*, it is conceivable that five-segmented tarsi already existed in the stem lineage of Dicondylia. Engel [18] even considered the five-segmented tarsus as the ancestral state of insects, with the number of tarsomeres being reduced not only in Euzygentoma, but also in Archaeognatha. However, it has to be emphasized that the number of tarsomeres is variable within Archaeognatha (2-3) and Zygentoma (2-4 in Euzygentoma; e.g. [46]).

Engel [18] proposed the dorsoventral flattening of the body as a synapomorphy uniting all Zygentoma. He classified *T. gertschi *in its own family Tricholepidiidae because the extinct type species of the Lepidotrichidae, *Lepidotrix pilifera *Silvestri, 1912, might be more closely related to the Euzygentoma than to *T. gertschi *due to the apparent absence of ocelli [17]. Engel [18] proposed the loss of ocelli as a synapomorphy uniting a clade "Neozygentoma" (= L. pilifera + Euzygentoma). Although *T. gertschi *clearly possesses three ocelli, we consider this a weak argument for this classification, since loss of ocelli occurred several times within Dicondylia, e.g. in Xenonomia (= Notoptera + Mantophasmatodea), Phasmatodea [51] and Zoraptera [52]. However, a more thorough re-examination of *L. pilifera *is mandatory. As for the head (judged from Silvestri's illustration of *L. pilifera*, his Figure V1 [53]) we particularly consider the corresponding, unique expression of the occiput as potentially synapomophic.

## Competing interests

The authors declare that they have no competing interests.

## Authors' contributions

AB, BW and BM designed the study, AB and FW conceived the experiments. AB and FW conducted the SRμCT, AB the SEM and phylogenetic analysis. AB, MK and BW carried out the analysis of the raw data. AB, MK, BW, and BM wrote the manuscript. All authors read, revised and approved the manuscript.

## Appendix 1

List of characters used for phylogenetic reconstruction

0. Orientation of head: (0) orthognathous; (1) prognathous or slightly inclined; (2) hypognathous

1. Number of ocelli: (0) 0; (1) 2; (2) 3.

2. Compound eyes: (0) composed out of more than 80 ommatidia; (1) less than 80 ommatidia;

3. Distance between eyes: (0) less than their own width; (1) greater than their own width; (2) eyes fused at single point; (3) eyes broadly fused along an eye seam

4. Shape of vertex: (0) flat, not developed into large protuberance; (1) conical, or developed into a large transverse ridge

5. Epicranial or coronal suture: (0) present; (1) absent

6. Parietal ridge: (0) absent; (1) present.

7. Postoccipital ridge: (0) present; (1) absent.

8. Subgenal ridge: (0) absent; (1) present

9. Pleurostomal ridge and circumocular ridge: (0) not in contact; (1) partly in contact

10. Interantennal ridge: (0) absent; (1) present

11. Shape of frons: (0) flat when seen from lateral; (1) outwardly bulged when seen from lateral

12. Distinct convexity ventrad the antennal bases: (0) absent; (1) present

13. Scutellum: (0) absent; (1) present

14. x-shaped median apodeme on the frontal region: (0) absent; (1) present

15. Clypeus: (0) not subdivided; (1) subdivided into ante- and postclypeus

16. Postclypeus: (0) not enlarged; (1) enlarged

17. Anteclypeus: (0) membranous; (1) sclerotised

18. Adult mouthparts: (0) with function; (1) without function

19. Oval sclerotization of labral base: (0) absent; (1) present.

20. Tormae: (1) absent; (0) present

21. Mesal extension of tormae: (0) present; (1) absent

22. M. epistoepipharyngealis (0 lb3): (0) present; (1) absent

23. M. labroepipharyngealis (0 lb5): (0) present; (1) absent

24. M. labrolabralis (0 lb6): (0) present; (1) absent

25. Insertion of antennae: (0) close to the anterior mandibular articulation with the pleurostomal and circumantennal ridges in contact (where applicable); (1) distinctly separated from the anterior mandibular articulation, pleurostomal and circumantennal ridges not in contact.

26. Antennifer: (0) present; (1) absent

27. Length of pedicel and scapus: (0) pedicel longer than scapus; (1) scapus longer than pedicel; (2) scapus and pedicel equal in length

28. Oval scerite in membrane connecting scapus and pedicellus: (0) absent; (1) present.

29. Size of first flagellomere: (0) not enlarged; (1) first flagellomere more than twice as long as second one.

30. Antennal stridulatory organ: (0) absent; (1) present

31. Areas of origin of antennal muscle 0an1: (0) anterior tentorial arms only; (1) anterior tentorial arms and tentorial bridge; (2) on dorsal tentorial arms only; (3) on dorsal arms and tentorial bridge; (4) anterior and dorsal tentorial arm.

32. Areas of origin of antennal muscle 0an2: (0) anterior tentorial arms only; (1) anterior tentorial arms and tentorial bridge; (2) on dorsal tentorial arms only; (3) on dorsal arms and tentorial bridge; (4) tentorial bridge only; (5) dorsal and anterior tentorial arms.

33. M. tentorioscapalis lateralis (0an3): (0) present; (1) absent

34. M. tentorioscapalis medialis (0an4): (0) present; (1) absent

35. Circumesophageal vessel ring branching off the dorsal aorta posterior to the brain: (0) present; (1) absent

36. Ostia of dorsal vessel: (0) lips always present; (1) ostia with and without lips (excurrent ostia).

37. Position and number of excurrent ostia within a segment: (0) one ventrolateral pair; (1) ventromedian.

38. Antennal circulatory organs in adults: (0) present; (1) absent

39. Antennal vessel wall: (0) uniform; (1) bipartite

40. Contractibility of antennal ampulla: (0) absent (non-pulsatile); (1) present (pulsatile).

41. M. interampullaris (0ah1): (0) absent; (1) present

42. M. ampulloaorticus (0ah2): (0) absent; (1) present

43. M. ampullopharyngealis (0ah3): (0) absent; (1) present

44. M. ampullo-frontalis (0ah4): (0) absent; (1) present

45. Connection of antennal ampulla to supraoesophageal ganglion: (0) absent; (1) present

46. Oval nuclei in tissue connecting the antennal ampulla and supraoesophageal ganglion: (0) absent; (1) present

47. Anterior and posterior tentoria: (0) seperated; (1) merged

48. Transverse mandibular tendon: (0) present; (1) absent

49. Processes of the anterior tentorial apodemes extending into the lumen of the mandibular base: (0) absent; (1) present

50. Corpotentorium: (1) elongated; (0) slim.

51. Apophyses on the anterior surface of the corpotentorium: (0) absent; (1) present

52. Secondary anterior tentorial bridge (“perforation of the corpotentorium"): (0) absent; (1) present.

53. Lateral lobes on the anterior tentorial arms: (0) absent; (1) present

54. Cuticular dorsal tentorial arms: (0) absent; (1) present

55. Trabeculae tentorii of posterior tentorial arms (0) present; (1) absent

56. M. tentoriofrontalis posterior (0te1): (0) present; (1) absent

57. M. posteriotentorialis (0te4): (0) present; (1) absent

58. M. tentoriotentorialis longus (0te5): (0) present; (1) absent

59. M. tentoriotentorialis brevis (0te6): (0) present; (1) absent

60. Numbers of incisivi on the left mandible: (0) 2; (1) 3; (2) 5; (3) 0; (4) 1; (5) 4

61. Numbers of incisivi on the right mandible: (0) 2; (1) 3; (2) 4; (3) 5; (4) 0; 5 (1)

62. Armament on the mesal side of the left mandible: (0) without teeth or ridges; (1) one tooth; (2) three ridges

63. Dorsal cutting edge of the left mandible: (0) notched; (1) smooth

64. Mandibular postmola: (0) absent; (1) present

65. Anterior mandibular joint: (0) absent; (1) present

66. Anterior mandibular joint: (0) cuticular hardening on the mandibular depression; (1) channel-joint (2) ball-and-socket joint

67. Anterolateral part of the anterior mandibular articulation (paratentorial joint): (0) present; (1) absent

68. Posterior mandibular joint: (0) cylinder-shaped (1) ball-and-socket joint

69. Mandibular ligament: (0) present; (1) absent.

70. M. craniomandibularis externus anterior (0md2): (0) present; (1) absent

71. M. hypopharyngomandibularis (0md4): (0) present; (1) absent

72. M. tentorio-mandibularis lateralis superior (0md5): (0) present; (1) absent

73. M. tentorio-mandibularis medialis superior (0md7): (0) present; (1) absent

74. Cardo: (0) present; (1) absent

75. Division of stipes into basistipes and mediastipes: (0) present; (1) absent

76. Galea: (0) present; (1) absent

77. Distal field of trichomes on the galea: (0) undivided; (1) divided; (2) just a U-shaped seam

78. Connection of lacinia and galea: (0) separated; (1) fused

79. Shape of lacinia: (0) sickle-shaped; (1) chisel-shaped; (2) truncate; (3) short claw

80. Mesally directed setae on lacinia: (0) present; (1) absent

81. Lacinia: (0) free; (1) in galeal cavity

82. Lacinial incisivi: (0) present; (1) absent

83. Number of incisivi on lacinia: (0) 3; (1) 2; (2) 1; (3) more than 3

84. Dentisetae on lacinia: (0) present; (1) absent

85. Proximal apodeme on the lacinia: (0) absent; (1) present

86. Galeolobulus: (0) absent; (1) present

87. Maxillary palpus: (0) 5-segmented; (1) 4-segmented; (2) 1-segmented; (3) 3-segmented; (4) 6-segmented; (5) 7-segmented

88. Orientation of maxillary palpi: (0) ventrally oriented; (1) anteriorly or dorsally directed

89. 0mx7: (0) present; (1) absent

90. M. palpopalpalis maxillae primus (0mx12): (0) present; (1) absent

91. Postmentum: (0) not subdivided; (1) subdivided into submentum and mentum

92. Angle between submentum and mentum: (0) less than 60° or absent; (1) more than 60°

93. Curvature of submentum: (0) absent; (1) curved in lateral view

94. Median longitudinal tunnel of labium: (0) absent; (1) present

95. Median cleft of prementum: (0) absent; (1) present

96. Labium: (0) paraglossa and glossa seperated; (1) paraglossa and glossae completly fused

97. Glossa: (0) present; (1) reduced

98. Number of glossae: (0) 2; (1) 1;

99. Number of paraglossae: (0) 2; (1) 1;

100. Shape of paraglossa: (0) cylindrical, as wide as thick; (1) flat, wider than thick; (2) palpus-like.

101. Relative length of paraglossae and glossae: (0) about equally long; (1) paraglossae twice as long or longer

102. Orientation of labial palpi: (0) anterior or lateral; (1) ventral or posterior

103. Number of labial palpomeres: (0) 3; (1) 1; (2) 2; (3) 4

104. Shape of labial palpi: (0) approximately round in cross section; (1) dorsoventrally flattened

105. Length of labial palpi: (0) longer than glossae; (1) about as long as the glossae

106. Moveable hooks of labial palpi: (0) absent; (1) present

107. M. postoccipitoglossalis medianus (0la1): (0) present; (1) absent

108. M. postoccipitoglossalis lateralis (0la2): (0) present; (1) absent

109. M. postoccipitoparaglossalis (0la3): (0) present; (1) absent

110. M. postoccipitoprementalis (0la4): (0) present; (1) absent

111. 0la5: (0) present; (1) absent

112. Origin of M. tentoriopraementalis inferior 0la5 (M.29): (0) ventral apodeme; (1) posterior tentorial arms; (2) posterior tentorial arms (posttentoria) and postocciput.

113. M. tentorioparaglossalis (0la6): (0) present; (1) absent

114. Origin of M. tentorioparaglossalis (0la6): (0): tentorium; (1) basal edge of prementum

115. M. tentorioglandularis (0la7): (0) present; (1) absent

116. M. submentopraementalis (0la8): (0) present; (1) absent

117. M. submentopraementalis (0la8): (0) one component; (1) two components

118. M. postmentomembranus (0la9): (0) present; (1) absent

119. M. submentomentalis (0la10): (0) absent; (1) present

120. M. praementoparaglossalis (0la11): (0) present; (1) absent

121. M. praementoglossalis (0la12): (0) present; (1) absent

122. M. praementopalpalis internus (0la13): (0) present; (1) absent

123. M. praementopalpalis externus (0la14): (0) present; (1) absent

124. Hypopharynx overlapping paraglossae and glossae 0) absent; 1) present

125. Shape of hypopharynx: (0) slope like; (1) distinctly flattened

126. Superlinguae: (0) present; (1) absent

127. Salivary glands and ductus: (0) present; (1) absent

128. Connection of salivary ducts: (0) connected before opening, Y-shaped; (1) open separately

129. M. frontobuccalis lateralis (0hy2): (0) present; (1) absent

130. M. craniohypopharyngealis (0hy3): (0) present; (1) absent

131. M. postmentoloralis (0hy6): (0) present; (1) absent

132. M. praementosalivaris posterior (0hy8): (0) absent; (1) present

133. M. lorosalivarialis (0hy11): (0) present; (1) absent

134. 0hy12: (0) present; (1) absent

135. M. frontobuccalis posterior (0bu3): (0) present; (1) absent

136. M. tentoriobuccalis lateralis (0bu4): (0) absent; (1) present

137. 0bu5: (0) present; (1) absent

138. M. tentoriobuccalis posterior (0bu6): (0) present; (1) absent.

139. Origin of M. tentoriobuccalis posterior 0bu6 (M.50): (0) anterior and/or posterior bridge, (1) pretentoria

## Supplementary Material

Additional file 1 **Homologization of the muscular terminology with other authors**. Abbreviations: +, muscle present (either in species described in the present study or the muscle is present in a study by a different author, but has no abbreviation); -, muscle absent;/, muscle not dealt with by the author; ?, unclear homology. Click here for file

Additional file 2 **Character state matrix in excel format together with short descriptions of the charactes used: (?) refers to a missing or unclear character state, (-) to inapplicable characters**. Click here for file

Additional file 3 **Character state matrix in nexus format: (?) refers to a missing or unclear character state, (-) to inapplicable characters**. Click here for file

Additional file 4 **3D model of the head of *T. gertschi***. Please use the open source software Blender (http://www.blender.org) to view the model. Click here for file

Additional file 5 **Image stack of transversal slices through the head of T. gertschi packed in the "avi" movie format for easy browsing through the stack**. Click here for file

Additional file 6 **Complete tree (strict consensus) showing all taxa**. Character numbers above, character states below the nodes. Click here for file
